# Increased Incidence of Depression and Chronic Pain in Traumatic Spinal Cord Injury Patients With Pre-Injury Alcohol Use Disorder: Longitudinal Analysis of Insurance Claim Database

**DOI:** 10.1089/neur.2023.0096

**Published:** 2024-01-12

**Authors:** Beatrice Ugiliweneza, Dengzhi Wang, Benjamin Rood, Maxwell Boakye, Camilo Castillo, Michal Hetman

**Affiliations:** ^1^ Kentucky Spinal Cord Injury Research Center, University of Louisville, Louisville, Kentucky, USA.; ^2^ Department of Neurological Surgery, University of Louisville, Louisville, Kentucky, USA.; ^3^ Department of Anatomical Sciences and Neurobiology, University of Louisville, Louisville, Kentucky, USA.; ^4^ Department of Pharmacology and Toxicology, University of Louisville School of Medicine, University of Louisville, Louisville, Kentucky, USA.; ^5^ Graduate Program in Biochemistry and Molecular Genetics, University of Louisville, Louisville, Kentucky, USA.

**Keywords:** addiction, alcohol use disorder, alcoholism, anxiety, depression, neurotrauma, outcome, pain, spinal cord injury

## Abstract

Alcohol use disorder (AUD) increases risk of traumatic spinal cord injury (SCI) and is associated with depression, anxiety, and chronic pain. Given that these neuropsychiatric morbidities are frequently observed in SCI patients, the effects of pre-injury AUD on risk of depression, anxiety, or chronic pain were analyzed using an insurance claim database. Of 10,591 traumatic SCI patients, 507 had AUD-associated claims in a 12-month period before injury. Those AUD-positive SCI patients showed distinct demographic characteristics, including greater representation of men, younger age, more comorbidities, lower coverage by commercial insurance, and more cervical-level injuries. The AUD group also showed elevated pre-injury comorbidity of depression, anxiety, and chronic pain. However, multi-regression analysis revealed an increased odds ratio (OR) of *de novo* diagnosis of post-SCI depression in AUD patients 6 months (1.671; 95% confidence interval [CI]: 1.124, 2.483) and 1 year post-injury (1.511; 95% CI: 1.071, 2.131). The OR of *de novo* post-SCI anxiety was unaffected by pre-injury AUD. Finally, 1 year after SCI, pre-injury AUD increased the OR of *de novo* diagnosis of post-injury chronic pain (1.545; 95% CI: 1.223, 1.951). Thus, pre-injury AUD may be a risk factor for development of depression and chronic pain after traumatic SCI.

## Introduction

Widely consumed and abused, alcohol is a major factor in increasing disease burden globally.^1^ However, only around one third of such an increase is attributable to conditions with a direct alcohol causation, including alcohol liver disease or alcohol withdrawal.^1^ The other portion of the alcohol disease burden is attributable to alcohol-mediated risk increase of various pathologies that may have other and/or additional causes.^1^ This category represents all types of traumatic injuries, including those related to motor vehicle accidents, sports, or occupation.^2,3^ In addition, chronic alcohol use disorder (AUD) is associated with several psychiatric/neurological comorbidities, including depression, anxiety, and chronic pain.^4–6^ Though the mechanistic causes of such associations are not entirely clear, AUD increases the risk of each of these conditions and vice versa.

Traumatic spinal cord injury (SCI) is a devastating disease that is associated with a large spectrum of long-term complications, including chronic pain and various mental health conditions.^7–9^ For instance, a longitudinal study of a large cohort of privately insured SCI patients versus non-SCI controls showed 47% or 69% higher risk for post-injury anxiety or mood disorders, respectively.^9^ In addition, a strong association between psychiatric morbidity and chronic pain was revealed in SCI patients.^9^ Therefore, chronic pain may play a causative role in declining mental health after SCI.^9^

Both acute alcohol consumption immediately before injury and pre-injury AUD are positively correlated with incidence of traumatic SCI.^10–14^ Such associations are not surprising given that alcohol increases risk of falls as well as motor vehicle or sports accidents, which may all lead to traumatic SCI.^2,3^ Whereas 24–49% of traumatic SCI patients screened positive for chronic, pre-injury AUD,^11–14^ only a few, relatively small, studies (*n* ≤ 155) addressed the effects of AUD on mental health complications of SCI. For instance, AUD versus non-AUD patients showed a similar incidence of depression.^15,16^ However, ongoing post-SCI AUD was linked to more depression, pain, and lower life satisfaction.^15,17^

The current study was initiated to investigate the effects of pre-injury AUD on chronic pain, anxiety, and depression using a large longitudinal sample of traumatic SCI patient records from an insurance claim database.

## Methods

### Data source

The records from 2000 to 2020 used for this study were accessed through the IBM MarketScan Research Database. The MarketScan is a healthcare research claims-based database that contains data for >265 million patients. It includes data from healthcare use over time tracked with claim codes along with demographics, insurer, and payments.^18^ We have a neurological and -surgical custom file with inpatient, outpatient, and prescription data. Each included person has a unique identifier that is used to link different services, allowing longitudinal health services research studies.

### Cohort selection

From the inpatient database, cases of traumatic SCI, ≥18 years of age, were extracted (see Supplementary Table S1 for International Classification of Diseases [ICD] code listing). A continuous enrollment for at least 12 months before injury (lookback period) was required to check for AUD codes (Supplementary Table S1). Pre-injury enrollment time was calculated as the difference between the coverage start enrollment date (or first claim in the data if that is missing) and injury hospitalization admission date. In addition, a post-injury continuous enrollment period of at least 12 months was required for outcome evaluation. Coverage end enrollment date (or last claim in the data if missing) and the injury hospitalization discharge date were used to calculate post-injury enrollment time.

### Study cohorts and comparisons

Twelve months of pre-injury claims were screened for AUD codes (see Supplementary Table S1). Those who did not have any AUD-associated claim were classified in the “non-AUD” category and compared to AUD-positive patients.

### Individual descriptors

Characteristics include demographics (age, sex), insurance type (commercial, Medicaid, or Medicare), and comorbidities (Elixhauser comorbidity score^19^ obtained using the adaptation to the International Classification of Diseases, Ninth Revision, Clinical Modification codes developed by Quan and colleagues^20^). SCI information available are injury level (cervical, thoracic, lumbar/sacral, and unknown) and injury type (non-traumatic and traumatic). Also included as a descriptor was the severity of injury through the International Classification of Diseases Injury Severity Score (ICISS).^21^

### Outcome of interest

Outcomes were evaluated at the initial SCI hospitalization and then at 6 and 12 months after hospital discharge. The following hospitalization outcomes were analyzed: length of acute post-injury hospital stay (not including inpatient rehabilitation); total payment; and discharge disposition. Post-discharge outcomes included depression, anxiety, and chronic pain (see Supplementary Table S1 for ICD code listing). In addition, healthcare utilization (emergency room visits, hospital readmissions, outpatient services, and outpatient medication refills) and associated payments were also analyzed. Payments were adjusted to 2020 U.S. dollars using the medical component of the Consumer Price Index (U.S. Bureau of Labour Statistics).

### Statistical analysis

Continuous variables were summarized with median and interquartile range (first to third quartiles) because they were not normally distributed per the Smirnov-Kolmogorov test and were compared with the Brown-Mood test. Categorical variables were summarized with counts and percentages and were compared with the chi-square test. To evaluate the effect of alcohol use on outcomes, quantile regression to the median was used for continuous variables and logistic regression was used for categorical variables. In these models, dependent variables (outcomes) were chronic pain, anxiety, or depression whereas all the individual characteristics (as listed in Table 1) and AUD were predictor variables. SCI patients with pre-injury positivity for the analyzed outcome were excluded from the analysis. For each analyzed outcome, pre-injury diagnosis of the other outcomes was included as an additional predictor variable (e.g., pre-injury chronic pain or anxiety when analyzing effects on post-injury, *de novo* depression). Continuous outcomes were summarized with regression-adjusted median and interquartile range (first to third quartiles), and categorical variables were summarized with regression-adjusted probabilities. All tests were two-sided with a significance level of 5%. Statistical analyses were performed in SAS software (version 9.4; SAS Institute Inc., Cary, NC).

**Table 1.** Demographic Characteristics of AUD and Non-AUD Patients

		**Non-AUD vs. AUD**		
		**Non-AUD**	**AUD**	*p* **value**
		**(** *n* ** = 10,084)**	**(** *n* ** = 507)**	
Age	Median [IQR]	55 [37–72]	51 [39–59]	**<0.0001**
Sex, female, *n* (%)		4854 (48)	135 (27)	**<0.0001**
Insurance	Commercial, *n* (%)	4947 (49)	203 (40)	
	Medicaid, *n* (%)	2279 (23)	259 (51)	**<0.0001**
	Medicare, *n* (%)	2858 (28)	45 (9)	
Elixhauser index	0, *n* (%)	3269 (32)	94 (19)	
	1, *n* (%)	3066 (30)	129 (25)	**<0.0001**
	2, *n* (%)	1961 (19)	124 (24)	
	3+, *n* (%)	1788 (18)	160 (32)	
Injury level	Cervical, *n* (%)	4245 (42)	302 (60)	
	Thoracic, *n* (%)	3341 (33)	122 (24)	**<0.0001**
	Lumbar/sacral, *n* (%)	2205 (22)	75 (15)	
	Unknown, *n* (%)	293 (3)	8 (2)	
ICISS	Median [IQR]	0.67 [0.55–0.79]	0.69 [0.55–0.82]	0.4666

AUD, alcohol use disorder; IQR, interquartile range; ICISS, International Classification Injury Severity Score.

## Results

### Different characteristics of traumatic spinal cord injury patients with a history of pre-injury alcohol use disorder

Analysis of the database records identified *n* = 10,591 SCI patients with data that covered a period from 1 year pre-injury to 1 year post-discharge (Table 1). In that group, 507 (4.79%) showed evidence of AUD before SCI, including insurance claim diagnoses of alcohol abuse (360; 3.4%), alcohol dependence (287; 2.71%), and/or unspecified use of alcohol (25 [0.24%]; Supplementary Table S2).

Importantly, SCI patients with versus without AUD showed significant differences in their demographic profiles, insurance status, number of comorbidities, and SCI type/level, but not injury severity (Table 1). The AUD group was younger (51 vs. 55, *p* < 0.0001), included less women (27% vs. 48%, *p* < 0.0001), and showed lower participation in both Medicare (9% vs. 28%) and private insurance (40% vs. 49%), but higher participation in Medicaid (51% vs. 23%, *p* < 0.0001; Table 1). Moreover, AUD patients showed a higher fraction of cervical-level SCI (60% vs. 42%, *p* < 0.0001; Table 1). In addition, as expected from widely recognized positive associations between AUD and chronic pain, anxiety, or depression,^4–6^ AUD versus non-AUD patients showed significantly higher incidence of those conditions before SCI (Table 2).

**Table 2.** Depression, Anxiety, and Chronic Pain Are Pre-Injury Comorbidities of AUD

**Outcomes**	**Non-AUD vs. AUD**		
	**Non-AUD**	**AUD**	*p* **value**
	**(** *n* ** = 10084)**	**(** *n* ** = 507)**	
12 months before SCI			
Depression alone, *n* (%)	688 (7)	100 (20)	**<0.0001**
Anxiety alone, *n* (%)	557 (6)	51 (10)	**<0.0001**
Depression and anxiety, *n* (%)	439 (4)	81 (16)	**<0.0001**
Chronic pain, *n* (%)	356 (4)	79 (16)	**<0.0001**

AUD, alcohol use disorder; SCI, spinal cord injury.

### Effects of pre-injury alcohol use disorder on post–spinal cord injury incidence of chronic pain, anxiety, and depression

Multi-variable regression models were used to determine the odds ratio (OR) of the incidence of chronic pain, anxiety, or depression at 6 or 12 months after discharge from the initial hospitalization attributable to SCI. For each of those outcomes, cases that showed positive pre-injury morbidity (Table 2) were excluded from the analysis.

**FIG. 1.**
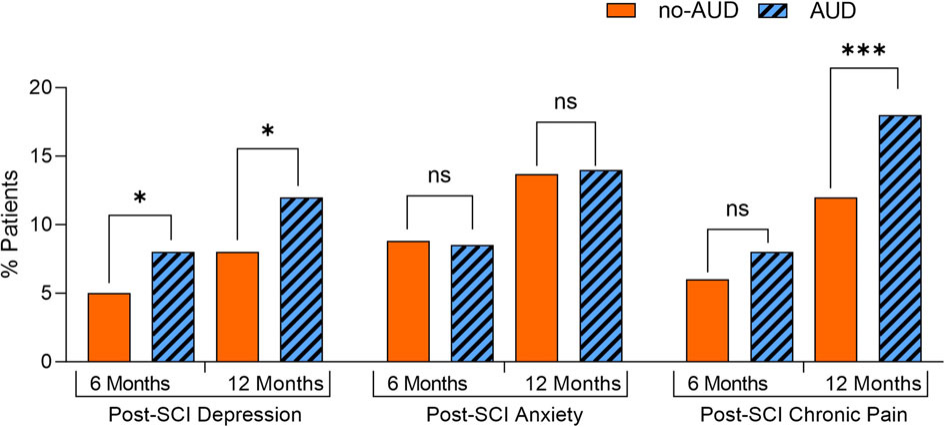
Effects of pre-injury AUD on incidence of depression, anxiety, and chronic pain after SCI. Multi-variable regression model adjusted fractions of SCI patients with *de novo* diagnosis of depression, anxiety, and chronic pain at 6 or 12 months after discharge are shown. Note significant increases of post-SCI depression and chronic pain in patients with pre-injury AUD. The post-discharge period started after the initial hospitalization attributable to SCI (median duration 7–8 days); *p < 0.05; ***p < 0.001; ns, *p* > 0.05, X^2^ test. AUD, alcohol use disorder; ns, not significant; SCI, spinal cord injury.

Importantly, pre-injury AUD was identified as an independent predictor of post-SCI depression at either 6 or 12 months after discharge (Table 3). AUD increased depression OR to 1.671 (95% CI: 1.124, 2.483) and 1.511 (95% CI: 1.071, 2.131) at 6 and 12 months, respectively. In addition, female sex, comorbidity index >0, pre-injury anxiety, insurance status (Medicaid vs. commercial), and injury severity were each associated with increased OR of post-SCI depression (Table 3). Conversely, lower depression OR was found in non-cervical SCI (each non-cervical category vs. cervical at 6 months; lumbar/sacral or unknown vs. cervical at 12 months; Table 3). After adjustment for such confounding effects, AUD versus non-AUD patients showed significantly increased incidence of post-SCI depression (8% vs. 5% or 12% vs. 8% at 6 or 12 months after discharge, respectively; *p* < 0.05; Fig. 1).

**Table 3.** Multi-Variable Regression Analysis of Effects by Pre-SCI AUD on Post-SCI Incidence of Depression, Anxiety, and Chronic Pain

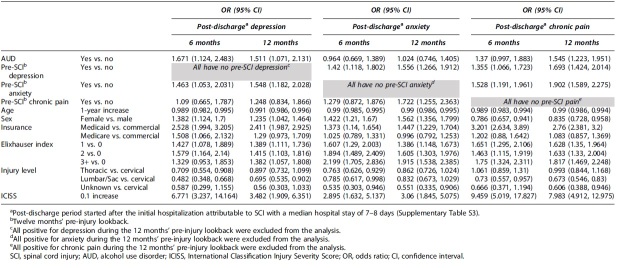

Female sex, comorbidity index >0, pre-injury depression, insurance status (Medicaid vs. commercial), and injury severity, but not AUD, were each associated with increased OR of post-SCI anxiety both 6 and 12 months after discharge (Table 3). At 12, but not 6, months after discharge, increased anxiety OR was also observed for patients with pre-injury chronic pain. Conversely, lower anxiety OR was associated with non-cervical SCI (Table 3). At 6 months after discharge, a significantly lower anxiety OR was found for each category of non-cervical SCI (Table 3). At 12 months, such an effect was only observed for unknown versus cervical-level injuries (Table 3). Consistent with non-significant effects of AUD on post-SCI anxiety, the incidence of this condition was similar in AUD versus non-AUD groups once adjusted for confounding factors (8.5% vs. 8.7% or 14% vs. 13.7% at 6 or 12 months after discharge, respectively; *p* > 0.05; Fig. 1).

Significantly increased OR of post-SCI chronic pain was observed in AUD versus non-AUD patients 12 months after discharge (1.545; 95% CI: 1.223, 1.951; Table 3). In addition, 6 months after discharge, there was a non-significant trend toward higher OR of chronic pain in the AUD group (1.37; 95% CI: 0.997, 1.883; Table 3). Other identified OR boosters of post-SCI chronic pain included pre-injury anxiety or depression, insurance status (Medicaid vs. commercial), and injury severity. Reduced chronic pain OR was associated with female sex and each category of non-cervical SCI (thoracic or lumbar or unknown vs. cervical-level SCI; Table 3). After adjustment for all confounding effects, significantly more AUD versus non-AUD patients were *de novo* diagnosed with chronic pain 12 months after discharge (18% vs. 12%, *p* < 0.05; Fig. 1). Non-significant differences in chronic pain were observed 6 months after discharge (8% vs. 6% in AUD vs. non-AUD patients; *p* = 0.0521; Fig. 1).

### Effects of pre-injury alcohol use disorder on outcomes of the initial spinal cord injury hospitalization and post-discharge healthcare utilization

Multi-variable regression-adjusted outcomes of the initial post-SCI hospitalization, including surgery but excluding inpatient rehabilitation, were compared in AUD versus non-AUD patients. Analyzed outcomes were length of stay, total cost, and percentage of patients discharged to their homes (Supplementary Table S3). Of those, only the total cost was significantly affected (median costs of $54,878 vs. $34,427 in the non-AUD vs. AUD group; *p* < 0.0001; Supplementary Table S3). The latter effect is unlikely to be related to different insurance coverage, type of SCI, or injury severity given that effects of such confounders were considered by the regression adjustments.

After multi-variable regression adjustments, AUD patients showed few subtle differences in healthcare utilization after discharge. Those include increased number of outpatient services (median of 109 vs. 95 for AUD vs. non-AUD 12 months after discharge; *p* = 0.0234; Supplementary Table S4). Additionally, total healthcare costs were lower at 6, but not 12, months after discharge in AUD versus non-AUD patients (Supplementary Table S4).

## Discussion

The current longitudinal analysis of the insurance claim database revealed that for at least up to 1 year after traumatic SCI, pre-injury AUD is an independent predictor of increased OR for post-SCI incidence of depression and chronic pain. Hence, the history of pre-injury AUD may increase the risk of developing post-SCI depression and chronic pain. To the best of our knowledge, this is the first analysis of a large cohort of SCI patients to document effects of past alcohol abuse on incidence of post-SCI complications. With a critical impact of depression and chronic pain on patients' quality of life,^22,23^ current findings flag persons with a history of pre-injury AUD as particularly vulnerable for development of those complications. In addition, given that both depression and chronic pain may each stimulate alcohol consumption,^4,6^ their timely diagnosis and effective treatment may help control AUD after SCI.

The current study has several limitations. First, accuracy of diagnoses in insurance claim databases cannot be verified. Second, AUD is often underdetected and healthcare providers may be reluctant to document a positive AUD diagnosis, especially in relatively moderate cases.^24^ In consequence, 1) this analysis may have been biased toward more severe AUD, and 2) the non-AUD group may have included those who were undiagnosed or whose AUD has not been documented and/or treated. Third, the insurance claim database does not allow us to address the effects of various patterns of AUD or alcohol doses. Moreover, no insight is provided into potential changes in AUD pattern after SCI. Given that patients can only be grouped as AUD positive or negative, it is not possible to differentiate between the effects of moderate versus severe alcohol abuse except for a separate analysis of SCI outcome in alcohol-dependent persons. Fourth, unlike data from SCI clinical trials such as the NACTN (North American Clinical Trials Network),^25^ the insurance claim database does not cover detailed injury severity information or the trajectory of post-injury recovery. Therefore, AUD impact on those important SCI parameters could not be analyzed.

Fifth, with a lookback period of 1 year, we cannot exclude a possibility that some patients who were included in the outcome analysis had earlier depression, anxiety, or chronic pain. Such a false negativity may be particularly prevalent in the AUD population given that depression, anxiety, and chronic pain are all AUD comorbidities. Re-emergence of those pre-existing conditions after SCI could have biased the data toward their greater incidence in the AUD group.

In addition, unequal access to healthcare and/or different rates of healthcare use could affect the number of insurance claims and potentially distort conclusions about morbidity. Although regression adjustments were used to normalize for demographic differences including type of insurance, it is possible that healthcare access and/or use was lower for AUD versus non-AUD patients. Specifically, weaker social support networks, reduced access to transportation, and lower compliance may have negative effects on healthcare access and use in AUD patients. Yet, analysis of post-discharge data does not support lower healthcare use in that group (Supplementary Table S4). On the contrary, some forms of healthcare utilization are increased in AUD patients, including number of outpatient services. Therefore, increased incidence of post-SCI depression or chronic pain is unlikely a result of poor healthcare access for AUD patients. Finally, one should acknowledge that the multi-variable regression models that were used to evaluate outcome incidence may have missed the effects of other important predictors such as family history or presence of specific pre-disposing conditions.

Although positive associations between AUD and depression have been shown after SCI,^26,27^ effects of pre-injury AUD on post-SCI depression have been rarely directly addressed. Small, prospective studies showed no significant changes in depression after SCI in pre-injury AUD patients.^15,16^ Reasons as to why the current analysis showed different effects may include a 1) larger, more representative sample size and/or 2) potential bias toward severe AUD given that moderate AUD is likely under-represented in the insurance claim database. Regardless, our longitudinal analysis of the insurance claim database provides a strong evidence to support a positive link between pre-injury AUD and depression.

Interestingly, analysis of 1035 SCI patient data from the multi-center Model System cohort showed increased risk of depression 5 years after SCI in patients who abstained from alcohol after being AUD positive at 1 year post-injury.^26^ Therefore, as shown in other AUD populations,^28^ SCI patients with a history of AUD may be particularly vulnerable to depression even after reducing alcohol use. Moreover, similarly to depression, chronic pain shows increased incidence and severity in abstainers with a history of AUD.^6^ Because the current data do not provide information about post-injury changes in AUD pattern, it is not possible to determine whether post-SCI abstinence may have played a role in increased depression or chronic pain in patients with pre-injury AUD. However, after traumatic SCI, many patients with pre-injury AUD reduce alcohol consumption at least during the first year post-trauma.^13^ Therefore, similarly to the reported effects in AUD patients who reduced/eliminated drinking,^6,26,28^ such a post-SCI abstinence may contribute to increases in depression and chronic pain in patients with a history of pre-injury AUD.

Positive associations between pre-injury AUD and post-injury depression and/or chronic pain have also been reported in other types of acute central nervous system (CNS) injury. A male-specific effect of high alcohol consumption was observed in a small longitudinal study of stroke patients (*n* = 191).^29^ Analysis of the insurance claim database found a moderate, yet significant, effect of AUD on post-stroke depression in a large sample of ischemic stroke patients (*n* > 170,000; hazard ratio, 1.08; 95% CI, 1.01–1.14).^30^ Interestingly, a longitudinal analysis of a relatively small clinical trial cohort (*n* = 283) showed increased depression severity 1 year after traumatic brain injury in patients with a history of AUD 1 year pre-injury.^31^ A similar approach, with a bigger sample (*n* = 559), revealed that pre-injury AUD was associated with a significant risk of transient, prolonged, or persistent post-TBI depression.^32^ A large observation cohort of 95,134 trauma patients showed that the OR of post-trauma chronic pain was 1.4 (95% CI, 1.2–1.7) in those with a history of alcoholism.^33^ However, such propain effects of pre-injury AUD may be dependent on the type of traumatic injury.^34^ Thus, a link between pre-injury AUD and post-injury depression and/or chronic pain is not unique to SCI.

What could be the potential mechanisms behind such an association? First, long-lasting alcohol abuse results in chronic systemic inflammation and neuroinflammation.^35^ Those, in turn, may contribute to depression as demonstrated both in rodent models and in humans.^36^ Whereas CNS injury is by itself a powerful stimulus for neuroinflammation, such a response could be further enhanced if the neuroinflammatory system has been primed by pre-injury AUD.^37^ Moreover, such priming could produce a long-lasting potentiation of neuroinflammation after chronic alcohol abuse has stopped. Second, depression is an AUD comorbidity and its higher post-SCI incidence in patients who are positive for pre-injury AUD may reflect increased depression potential in alcohol abusers.^4,28^ Similar mechanisms may also mediate the AUD-associated increase in post-SCI chronic pain.^6^ AUD is a well-established cause of a painful peripheral neuropathy.^38^ Although its driving mechanisms are not entirely clear, neuroinflammation at the spinal cord level appears to be one of its correlates.^38,39^ In addition, comorbidity of AUD and chronic pain^6^ suggests that a subset of SCI patients with pre-injury AUD may be more susceptible to develop chronic pain post-injury.

Taken together, increased post-SCI depression and chronic pain may have significant negative impact on post-injury recovery and quality of life of patients with pre-injury AUD. Given that pre-injury AUD lowers participation and efficacy of post-SCI rehabilitation,^40,41^ timely detection and treatment of depression and chronic pain may improve those outcomes. Future studies are needed to determine whether positive interactions between AUD and post-injury depression/chronic pain are dependent on AUD pattern/severity, sex, and/or other substance abuse. Identifying the mechanisms of those interactions could help optimize treatment, including consideration for the various anti-inflammatory strategies that are being developed for depression.^36^ Given that lower socioeconomic status of many AUD patients may further magnify negative consequences of SCI, identifying and treating post-SCI complications that are frequent in that population is of great importance to alleviate the burden of SCI on persons, their families, and society.

## Supplementary Material

Supplemental data

Supplemental data

Supplemental data

Supplemental data

## Transparency, Rigor, and Reproducibility Summary

All deidentified data used in this analysis are available in the insurance claim database from a commercial vendor (IBM MarketScan Research Database). All methodological aspects of the analysis were described in the methods with diagnostic codes that were included in the analysis listed in Supplementary Table S1. The sample included 10,591 cases of traumatic SCI. The codes used for the analysis will be shared by the authors upon a reasonable request.

## Abbreviations Used

AUD: alcohol use disorder
CI: confidence interval
CNS: central nervous system
ICD: International Classification of Diseases
OR: odds ratio
SCI: spinal cord injury
